# Broad PFAS Binding with Fatty Acid Binding Protein 4 Is Enabled by Variable Binding Modes

**DOI:** 10.1021/jacsau.5c00504

**Published:** 2025-06-02

**Authors:** Aaron S. Birchfield, Faik N. Musayev, Abdul J. Castillo, George Zorn, Brian Fuglestad

**Affiliations:** † Department of Chemistry, 6889 Virginia Commonwealth University, Richmond, Virginia 23284, United States; ‡ Department of Medicinal Chemistry, School of Pharmacy, Virginia Commonwealth University, Richmond, Virginia 23298, United States; § The Center for Drug Discovery, Virginia Commonwealth University, Richmond, Virginia 23298, United States

**Keywords:** Fatty acid binding protein 4, FABP4, per- and polyfluoroalkyl substances, PFAS, fluorinated ligands, protein structure, X-ray crystallography

## Abstract

Per- and polyfluoroalkyl substances (PFAS) are ubiquitous pollutants that bioaccumulate in wildlife and humans, yet the molecular basis of their protein interactions remains poorly understood. Here, we show that human adipocyte fatty acid-binding protein 4 (FABP4) can bind a diverse array of PFAS, including next-generation replacements for legacy chemicals and longer-chain perfluorocarboxylic acids. Shorter-chain PFAS, although weaker binders, still displayed measurable affinities, surpassing those of their nonfluorinated analogs. We determined crystal structures of FABP4 bound to perfluorooctanoic acid (PFOA), perfluorodecanoic acid (PFDA), and perfluorohexadecanoic acid (PFHxDA), revealing three distinct binding modes. Notably, PFOA binds in two separate sites, and two distinct conformations define single-ligand binding of PFDA and PFHxDA. These arrangements enhance hydrophobic interactions within the binding cavity and likely explain the low micromolar dissociation constants observed in fluorescence competition assays. Our findings underscore the critical roles of chain length, headgroup functionality, and protein conformation in PFAS–FABP4 interactions. Given the emerging implications of the role of FABP4 in endocrine function, even subtle PFAS-induced perturbations could affect metabolic regulation and disease risk. Overall, this work highlights the value of direct structural and biochemical insights into PFAS–FABP4 interactions and paves the way for future research on PFAS transport and toxicological outcomes.

## Graphical abstract

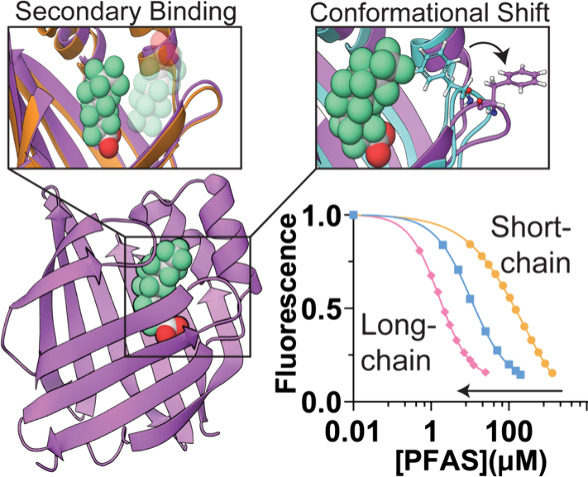

Per- and polyfluoroalkyl substances (PFAS) are a diverse class of synthetic chemicals characterized by fully or partially fluorinated carbon chains, which confer exceptional chemical and thermal stability, making them resistant to environmental degradation.^1,2^ These properties have led to widespread PFAS use in industrial and consumer products such as nonstick cookware, stain-resistant textiles, firefighting foams, and food packaging.^3^ Decades of use have produced global environmental distribution and measurable levels in wildlife and humans.^4,5^ Understanding PFAS bioaccumulation, transport, and protein interactions is critical for assessing health risks. Due to their chemical and physical similarity to lipids, PFAS have a propensity to interact with lipid carrier and lipid binding proteins, which are emerging as important targets to understand the health effects of PFAS exposure.^6–9^ To date, detailed structural investigation of PFAS protein binding has mostly focused on peroxisome proliferator-activated receptors (PPARs),^10–12^ human serum albumin (HSA),^13^ liver fatty acid-binding protein (FABP1),^14–16^ and transthyretin.^17,18^ All of these proteins are known lipid binders, leaving the role of other potential lipid binding protein interactions underexplored. Despite great importance, at the time of writing, there exist only 11 experimentally determined PFAS bound structures available on the Protein Data Bank (PDB) (Table S1), which significantly limits understanding of the molecular drivers of PFAS-protein interactions.

The interactions of PFAS with FABP1 have been extensively studied.^14,15,19^ Interest in exploring this FABP isoform stems from the known accumulation of PFAS in the liver.^20–23^ However, it is possible that other FABP isoforms are likely effective binders of PFAS and responsible for bioaccumulation and health effects.^6–9,24,25^ Aside from liver and kidneys, PFAS are known to accumulate in circulation.^26,27^ While adipocyte fatty acid-binding protein (FABP4) is the only known secreted, circulatory fatty acid-binding protein, it has garnered surprisingly little attention in PFAS research, with an unrecognized role in human health effects from PFAS exposure. It transports fatty acids in adipocytes,^28–30^ macrophages,^31–33^ and endothelial cells^28,34,35^ and was recently shown to regulate beta-cell function via the Fabkin hormone complex, influencing insulin secretion and metabolic homeostasis.^36^ While the role of ligand binding to FABP4 within the Fabkin complex has yet to be elucidated, potential for modulation by circulatory PFAS may account for observed health effects.^9,37,38^ Elucidating the binding interactions between FABP4 and PFAS could provide new insights into biological transport and health effects of this interaction.

While PFAS such as perfluorooctanoic acid (PFOA) and perfluorooctanesulfonic acid (PFOS) have been phased out due to regulatory actions, persistence in environmental reservoirs necessitates investigation into human health effects.^39^ Numerous replacement PFAS compounds, such as GenX, ADONA, and F-53B, are being introduced.^40,41^ Computational studies have assessed binding and bioaccumulation of these compounds in HSA.^42,43^ However, experimental data on their interactions with other proteins, and on their persistence and toxicity, remain limited. A wide-ranging understanding of protein binding to both legacy and emerging PFAS is thus essential to predict transport, accumulation patterns, and health effects across different classes of PFAS.

Here, we present the first comprehensive experimental study of PFAS interactions with FABP4. We measured the binding affinity for various PFAS by displacement of the fluorescent probe 8-anilinonaphthalene-1-sulfonic acid (ANS), establishing a relationship between chemical features of PFAS and their ability to bind with FABP4. We solved crystal structures of FABP4 bound to PFOA (PDB: 9MIW), perfluorodecanoic acid (PFDA) (PDB: 9MIZ), and perfluorohexadecanoic acid (PFHxDA) (PDB: 9MP2), revealing distinct binding modes for each PFAS. These findings provide novel insights into the molecular mechanisms of PFAS binding to FABP4, which has high potential exposure to PFAS in its circulatory form. This study lays a foundation for understanding protein-driven PFAS bioaccumulation, transport, and toxicity in circulation.

Because FABP4 purified from *E. coli* is known to retain endogenous lipids^44^ that are challenging to remove,^45^ a butanol-extraction method was developed to achieve full delipidation at high yield (details in the Materials and Methods and Figure S1 in the Supporting Information). A fully apo, functional FABP4 was confirmed by crystallization after the delipidation procedure (PDB: 9OB7) and by observation of palmitic acid binding by protein NMR, which produced a spectrum nearly identical to FABP4 containing endogenous lipids (Figure S1). To understand the potential for PFAS interactions with FABP4, a comprehensive set of binding affinity measurements was obtained using an ANS displacement assay for a structurally diverse range of PFAS and alkanoic acid analogs of perfluorocarboxylic acids (PFCAs) (Figure 1, Figures S2–S5, Table S2). ANS was confirmed to bind to the canonical lipid binding site through crystallography, indicating that it is an accurate displacement probe for PFAS binding to the central hydrophobic cavity (PDB: 9OB8, Figure S2B). The results revealed clear evidence that the binding affinities of PFCAs are markedly dependent on chain length. Longer-chain PFCAs exhibit substantially higher binding affinities, as illustrated by the low micromolar K_d_ values of perfluorotetradecanoic acid (PFTeDA), perfluorotridecanoic acid (PFTrDA), and perfluorododecanoic acid (PFDoA), which all display K_d_ values below 1 μM. These findings are consistent with previous reports that have shown increased protein binding affinity with increasing PFAS chain length across various protein targets, including serum albumin and liver FABPs.^15,16,19,46^ This trend reflects the greater hydrophobic surface area and enhanced van der Waals interactions afforded by longer perfluorinated chains. Notably, this trend closely parallels the behavior of naturally occurring long-chain saturated fatty acids, such as palmitic acid, which display similar low micromolar K_d_ values.^47,48^

**Figure 1:**
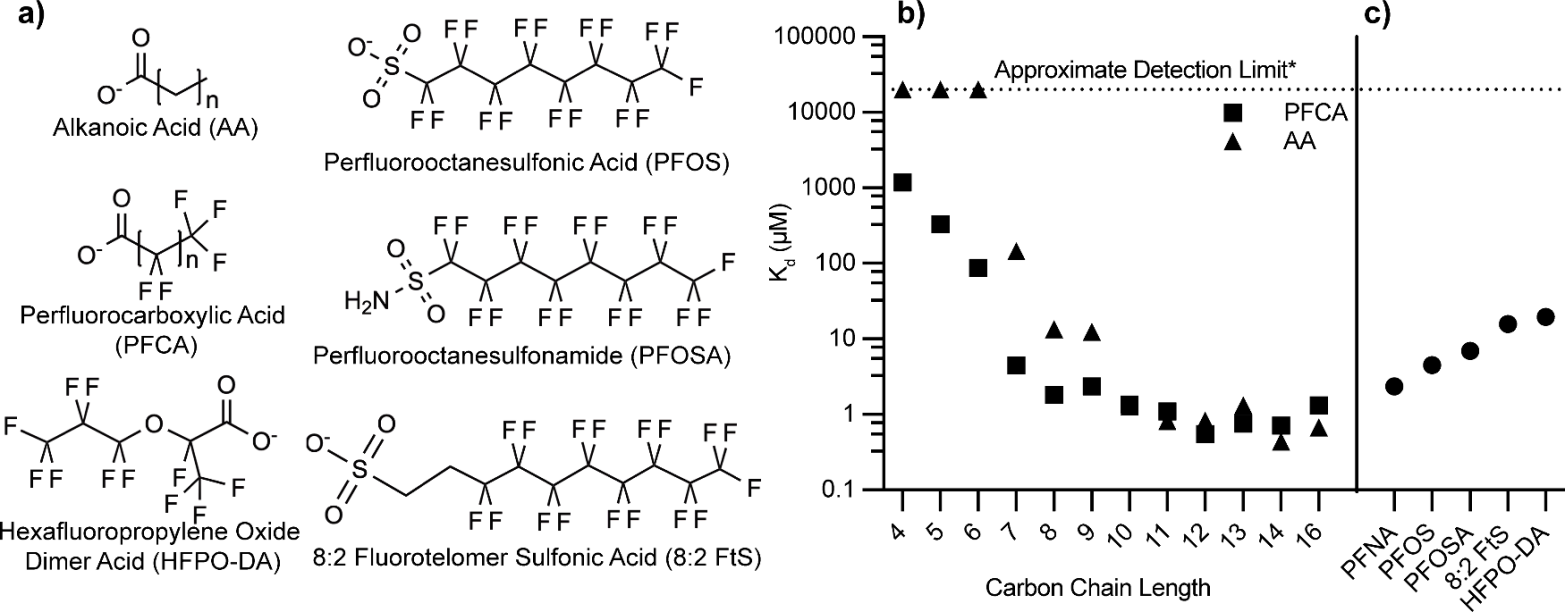
Structures and FABP4 binding affinities of compounds examined in this study. (a) Chemical structures of PFAS and alkanoic acid compounds that were measured for affinity to FABP4. (b) Binding affinity (K_d_) of perfluorocarboxylic acids compounds and their alkanoic acid analogs with FABP4. (c) Affinity of PFAS compounds analogous to perfluorononanoic acid (PFNA) with alternate headgroups to FABP4. K_d_ values were converted from IC_50_ measurements obtained from ANS displacement assays. Error bars are smaller than the diameter of the symbols. See Table S2 for detailed values including errors.

In contrast, shorter-chain PFCAs (e.g., perfluorohexanoic acid (PFHxA); perfluoropentanoic acid (PFPeA), perfluorobutanoic acid, PFBA) exhibited significantly weaker binding, with K_d_ values rising into the tens to hundreds of micromolar range. (Figure 1B, Figure S4, Table S2). This loss in binding strength can be attributed to diminished hydrophobic interactions and less extensive contacts formed within the FABP4 binding cavity. Nevertheless, even these shorter-chain PFAS exhibit measurable binding, which is especially pronounced in comparison to their nonfluorinated counterparts (Figure S3). These findings underscore a role for fluorination in stabilizing ligand-protein interactions, likely through enhanced hydrophobicity,^49^ even when hydrophobic chain length is insufficient to achieve strong binding in hydrogenated analogs.

In addition to chain length and the degree of fluorination, headgroup functionality exerts an influence on FABP4 binding affinity. This effect is evident when comparing perfluorononanoic acid (PFNA), PFOS, perfluorooctanesulfonamide (PFOSA), and 8:2 fluorotelomer sulfonic acid (8:2 FtS) (Figures S4 and S5), each having eight fluorinated carbons, while 8:2 FtS has a longer chain, with ten carbons total, eight of which are fully fluorinated. Despite sharing a similarity in the number of fluorinated carbons, there are differences in binding affinity. PFNA binds more tightly to FABP4 than PFOS, PFOSA, or 8:2 FtS, which suggests that sulfonate and sulfonamide headgroups slightly diminish affinity by interfering with stabilizing contacts typically achieved by carboxylate-containing ligands. Furthermore, the extended chain length of 8:2 FtS does not appear to confer any binding advantage, underscoring that headgroup identity and fluorination pattern are more critical determinants of affinity than additional methylene units. Together, these observations highlight the interplay between chain architecture, fluorination, and headgroup chemistry in governing PFAS-FABP4 interactions.

X-ray crystallographic analysis of FABP4 binding with PFOA, PFDA, and PFHxDA show distinct interactions that highlight the influence of chain length and fluorination on binding affinity (Figure 2 and Table S3). Notably, PFOA was found to bind in two distinct sites within FABP4 (Figure 2A). At the “primary site”, the carboxylate group is stabilized through hydrogen bonding with Arg 126 and Tyr 128, as well as a water-mediated hydrogen bond with Arg 106. The residues, Ala75, Thr29, Ala33, and Phe16 primarily interact through their hydrocarbon groups, contributing to hydrophobic interactions. PFOA binding at an observed ‘secondary site’ is stabilized by hydrogen bonding between its carboxylate group and Thr29 (Figure 2A and Figure S6). We note here that the assay likely reports only on the affinity of PFOA for binding to the primary site, due to the positioning of ANS here (Figure S2B).^50^ Since the X-ray electron density is less intense for the PFOA in the secondary site, we expect that binding is weaker here than for the primary site (Figure S7).

**Figure 2:**
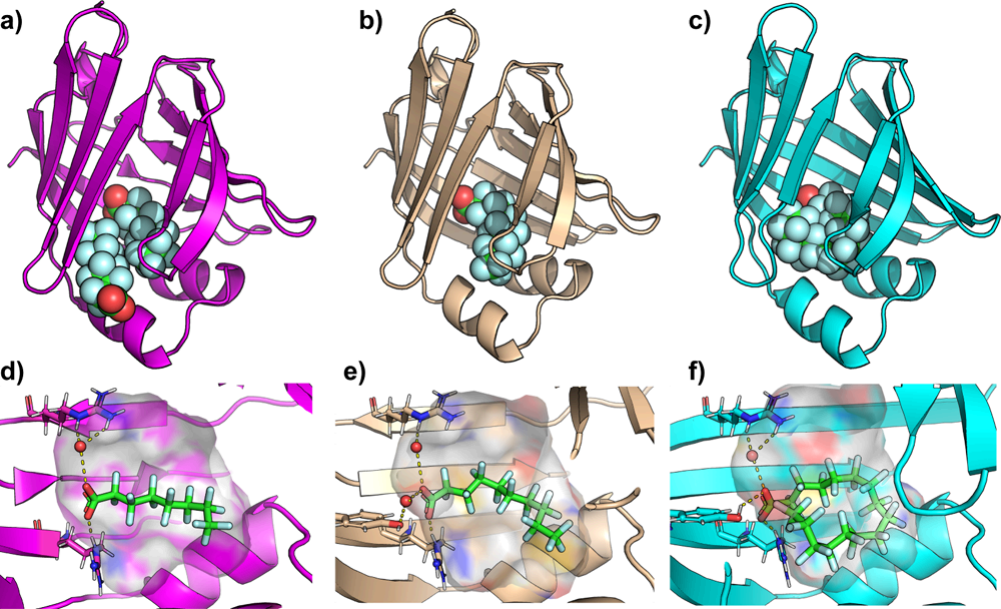
Crystal structures of PFOA, PFDA, and PFHxDA bound to FAPB4. (a) X-ray crystallographic structure of PFOA (spheres) bound to FABP4 (magenta cartoon), displaying two PFOA molecules bound. (PDB: 9MIW). (b) X-ray crystallographic structure of FABP4 (wheat cartoon) with a single PFDA bound (PDB: 9MIZ). (c) X-ray crystallographic structure of FABP4 (cyan cartoon) with a single PFHxDA bound (PDB: 9MP2). Zoomed images of the (d) “primary” PFOA, (e) PFDA, or (f) PFHxDA bound to the hydrophobic ligand binding cavity. The protein cavity is depicted as a semitransparent surface, colored according to atomic identity: gray for hydrogens, blue for nitrogens, and red for oxygens, with carbons colored according to the cartoon coloring scheme. PFOA, PFNA, and PFHxDA are depicted in sticks, with key hydrogen-bonds and charge interactions highlighted in dashed yellow lines. For clarity, the water mediated H-bond between PFDA and S53 is omitted in (e). Resolved water molecules that mediate PFAS–protein interactions are depicted in red spheres.

The binding pose of PFDA resembles that of the PFOA observed in the primary site (Figure 2, Figure S8). In the PFDA structure, however, the extended fluorinated chain allows closer interactions with residues, Ala75, Asp76, Thr29, Ala33, and Phe16, reducing the distance between these residues and the ligand by approximately 1 Å compared to PFOA. Both PFOA and PFDA induce an “open” conformation of Phe57, which results in a larger hydrophobic cavity, compared to the closed conformation of Phe57 in the apo structure (Figure 3A–C). Interestingly, this residue is implicated in access of lipids to the hydrophobic cavity.^44,51,52^ The open conformation of Phe57 is necessary to accommodate the secondary PFOA ligand and the terminal fluoromethyl of PFDA. The extended tail length of PFDA requires a change in conformation compared to the apo state, which suggests that the ten-carbon chain induces energetically unfavorable steric clashes with the edge of the hydrophobic cavity. The small changes in PFDA proximity to cavity residues likely enhance hydrophobic interactions and van der Waals forces, compensating for the energetic cost of inducing a conformational change. We note that a similar conformational change is observed with PFOA but is induced by the presence of a second ligand. The PFHxDA crystal structure highlights distinct conformational features that accommodate its extended fluorinated chain. While PFDA remains fully extended, PFHxDA adopts a U-shaped conformation beginning around carbons 8–10, with carbon 9 forming the bend’s apex (Figure 3d). This observation aligns with an *in-silico* analysis of rat FABP1, which suggested that PFCAs longer than ∼11 carbons must bend to fit within the binding pocket^16^ and resembles the binding mode of the hydrogenated analog, palmitic acid,^44^ a known natural ligand. Notably, Phe57 orients its aromatic ring inward in the PFHxDA-bound structure, resembling the closed conformation observed in the apo-FABP4, thereby enabling hydrophobic interactions (Figure 3).

**Figure 3:**
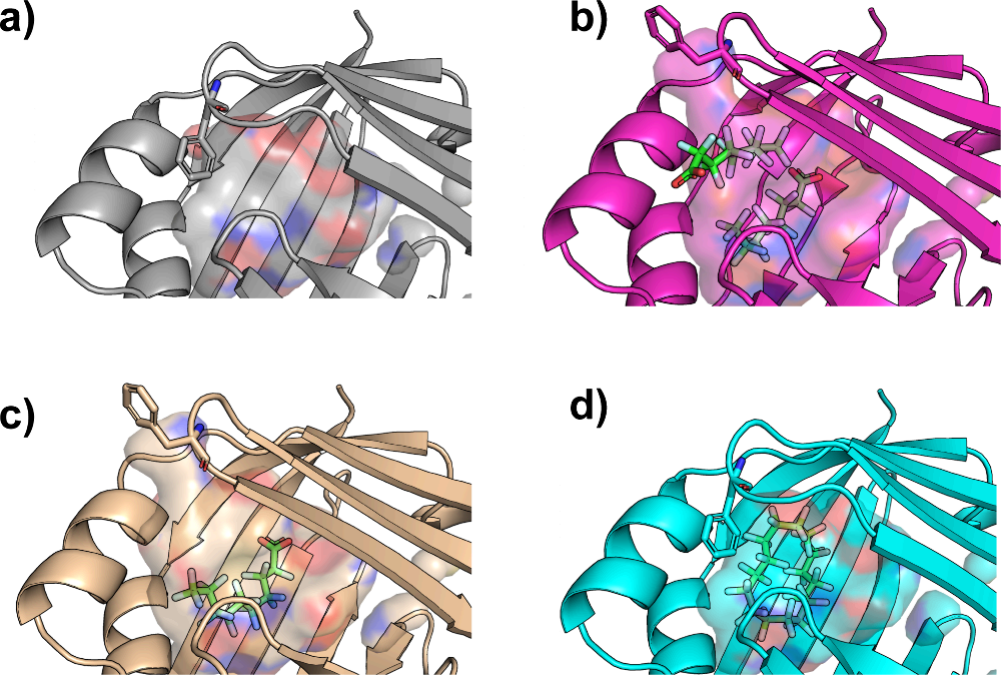
Phe57 changes conformation to accommodate PFAS ligands. Zoom of the region surrounding Phe57 in the crystal structures of (a) apo-FABP4 (PDB: 9OB7), (b) PFOA-bound FABP4 (PDB: 9MIW), (c) PFDA-bound FABP4 (PDB: 9MIZ), and (d) PFHxDA-bound FABP4 (PDB: 9MP2). Phe57 and the PFAS ligands are displayed as sticks and the ligand binding cavity is displayed as a semitransparent surface.

The crystal structures of PFOA, PFDA, and PFHxDA bound to FABP4 illustrate three distinct binding modes that underscore the protein’s adaptability to varying PFAS chain lengths. First, the PFOA-bound structure reveals two binding sites that accommodate the shorter-chain PFAS. Second, the PFDA-bound structure showcases a more extended perfluorocarbon tail, which induces an outward-facing Phe57, thereby creating sufficient space within the binding pocket. Third, the PFHxDA-bound structure adopts a U-shaped conformation, shifting Phe57 inward to maximize hydrophobic contacts. These observations, combined with our binding affinity data, indicate that while FABP4 can accommodate a range of PFCAs, those with longer chains must adopt a bent conformation to fit within the binding pocket. Inducing this degree of bending in a perfluorinated carbon chain requires more energy than for the corresponding hydrogenated carbon chain,^16^ which may be compensated for by the enhanced hydrophobicity from perfluorination compared to hydrogenated counterparts. Our crystal structures reveal non-native binding modes for PFAS that deviate from typical fatty acid interactions, suggesting that other proteins may likewise bind PFAS in previously unobserved modes. Notably, a recent *in silico* docking study concluded that FABP4 cannot bind longer PFCAs,^24^ illustrating the complexity of predicting PFAS interactions based on current structures and the critical need for experimental data. Additionally, the structural consequences of PFAS binding reported here may support PFAS-binding protein design efforts.^54^

Our results indicate that FABP4 binds to a range of PFAS. This raises the possibility that PFAS exposure could, even at low levels, interfere with normal FABP4 function in lipid signaling and metabolism. The results suggest that free FABP4 in circulation and bound within the Fabkin hormone complex may be susceptible to modulation by PFAS, hinting at novel mechanistic links between PFAS exposure and disease. Future studies that evaluate the downstream consequences of PFAS–FABP4 binding, particularly under varying exposure scenarios, will be critical for understanding how these interactions might contribute to metabolic dysregulation or disease risk. These insights strengthen our grasp of PFAS–protein interaction mechanisms and lay important groundwork for further investigations into their systemic health impacts.

## Supplementary Material



## Notes

Experimental procedures, materials and methods, including protein expression and purification, FABP4 delipidation, ANS competition assay, protein crystallization, and X-ray data collection and refinement, supplemental tables of PFAS–FABP4 binding affinities and X-ray crystal data statistics, and supplemental figures including NMR spectra, binding curves, electron density maps, and ligand interaction diagrams (PDF)
