# Supplementary material for: Broad PFAS Binding with Fatty Acid Binding Protein 4 Is Enabled by Variable Binding Modes
Source: JACS Au. 2025 Jun 2;5(6):2469–74. doi: 10.1021/jacsau.5c00504 (PMC12188406; doi:10.1021/jacsau.5c00504)
Supplement: Supplementary file 1 [file au5c00504_si_001.pdf]

Supporting Information:

Broad PFAS binding with fatty acid binding protein 4 is enabled by variable binding modes.

Aaron S. Birchfield[a], Faik N. Musayev[b,c], Abdul J. Castillo[a], George Zorn[a], Brian Fuglestad[a,c*].

[a] Department of Chemistry, Virginia Commonwealth University, Richmond, VA 23284, U.S.A.

[b] Department of Medicinal Chemistry, School of Pharmacy, Virginia Commonwealth University, Richmond, VA 23298, USA.

[c] The Center for Drug Discovery, Virginia Commonwealth University, Richmond, VA 23298, USA.

[*] To whom correspondence should be addressed: fuglestadb@vcu.edu

Materials and Methods

Chemicals and Materials

Heptafluorobutyric Acid (Perfluorobutanoic Acid) was obtained from Sigma-Aldrich. Perfluoropentanoic Acid (PFPeA), Perfluorohexanoic Acid (PFHxA), Perfluorononanoic Acid (PFNA), Perfluorotetradecanoic Acid (PFTeDA), Perfluorotridecanoic Acid (PFTrDA), Perfluorododecanoic Acid (PFDoA), Perfluorodecanoic Acid (PFDA), Hexafluoropropylene Oxide Dimer Acid (HFPO-DA), Perfluorohexanesulfonamide (PFOSA), and Perfluorooctanesulfonic Acid (PFOS) were purchased from Cayman Chemical. Perfluoroheptanoic Acid (PFHpA) and Perfluorooctanoic Acid (PFOA) were obtained from Sigma-Aldrich. Perfluorohexadecanoic Acid (PFHxDA) was acquired from Combi-Blocks.

Butanoic Acid, Hexanoic Acid, Heptanoic Acid, Octanoic Acid, Nonanoic Acid, Decanoic Acid, Dodecanoic Acid, Tetradecanoic Acid, and Hexadecanoic Acid were obtained from Sigma-Aldrich. Pentanoic Acid was sourced from Thermo Scientific. Undecanoic Acid and Tridecanoic Acid were acquired from Cayman Chemical. All other chemicals not explicitly mentioned in the text were purchased from commercial suppliers and used as received.

Protein Expression and Purification

Human Fatty Acid Binding Protein (FABP4) (Uniprot Accession: P15090) was recombinantly expressed in BL21(DE3) cells transformed with a pET-28a(+)-TEV vector and cultured in 2x M9 media as described in Azatian et al.[1], with the exception that BL21(DE3) cells were used instead of C41(DE3), and the media was supplemented with 100 µM $CaCl_2$, 35.96 µM $FeSO_4 \cdot 7H_2O$, and 29.65 µM thiamine hydrochloride in place of the original trace metals and biotin/thiamine cocktail. Cells were grown to an OD_{600} of 0.8-1 and induced with 1 mM IPTG for 16-18 hrs at 17°C. Cells were pelleted by centrifugation at 5000 × g for 30 minutes at 4°C, then stored at -80°C or used immediately. Pelleted cells were resuspended in Lysis Buffer (50 mM Tris-Cl pH 7.4, 300 mM NaCl, 2 mM Dithiothreitol (DTT), 0.5% Triton X-100) containing cOmplete™ Mini EDTA-free Protease Inhibitor Cocktail (Millipore Sigma). Lysozyme (Gold Bio) was added to a final concentration of 0.2 mg/mL, and the suspension was incubated on ice for 30-90 minutes.

Cells were sonicated and lysate centrifuged at 13,000 × g for 30 minutes at 4°C. The supernatant was collected, and imidazole was added to a final concentration of 10 mM. The mixture was applied to a 2 mL nickel-nitrilotriacetic acid (Ni-NTA) resin column (Gold Bio) pre equilibrated with Equilibration Buffer (50 mM Tris-Cl pH 7.4, 300 mM NaCl, 10 mM imidazole, and 2 mM DTT) using gravity flow. After collecting the flow-through, the resin was washed with 10 column volumes of Wash Buffer (50 mM Tris-Cl pH 7.4, 300 mM NaCl, 10 mM imidazole, and 2 mM DTT), or until protein concentration in the

flow-through was negligible as determined by Bradford assay. FABP4 was eluted with Elution Buffer (50 mM Tris-Cl pH 7.4, 300 mM NaCl, 300 mM imidazole, and 2 mM DTT) and desalted using a PD-10 column equilibrated with 20 mM Tris-Cl pH 7.4, 100 mM NaCl, 2 mM DTT, and 0.5 mM EDTA.

Recombinant Tobacco Etch Virus (TEV) protease, expressed and purified in-house, was added to the desalted eluate at a ratio of 30 μg TEV protease per 1 mg FABP4 (determined by Bradford assay). Proteolytic digestion was performed overnight at 4°C. The digested sample was then applied to a fresh 2 mL Ni-NTA resin column, and the flow-through containing cleaved FABP4 was collected. The resin was washed with 5 mL of Equilibration Buffer and the wash fractions were combined with the flow-through. The purified, digested FABP4 sample was dialyzed overnight at 4°C against 1 L of 20 mM Tris-Cl pH 7.4, 100 mM NaCl, 2 mM DTT, and 0.5 mM EDTA. The dialyzed sample was concentrated to approximately 1mg/mL in preparation for delipidation. Bradford assay measurements indicated typical yields of approximately 30 mg of FABP4 per 1L of culture, with SDS-PAGE analysis confirming the sample to be ≥95% homogeneous.

FABP4 Delipidation

FABP4 purified from *E. coli* is known to retain endogenous lipids,[2] which necessitates delipidation to prevent interference with downstream experiments. Previous studies have noted the difficulty of achieving complete delipidation of FABP4.[3] In our hands, methods such as resin-based extraction or guanidinium-induced denaturation and refolding resulted in ≥90% protein loss, yielding only 1–2 mg of purified protein. To address this, we developed a butanol-extraction method that achieved full delipidation while consistently yielding 10–12 mg of FABP4.

To delipidate the protein sample, n-butanol was used in a multi-step extraction process. Initially, 20% (v/v) n-butanol was added to the protein solution, which was then gently swirled and centrifuged at 4000 × g for 15 minutes at room temperature. The organic layer and interphase were carefully removed. This process was repeated twice using 15% (v/v) n-butanol, with centrifugation at 4000 × g for 10 minutes between each extraction. The organic layer and interphase were removed after each centrifugation. The interphase thickness was observed to decrease progressively with successive extractions. The solution was passed through a 0.22 μm filter to remove precipitates and lipid-protein complexes. Two additional extractions with 15% (v/v) n-butanol were performed, as described above, followed by a second filtration through a 0.22 μm filter. After this step, the interphase boundary between the water and n-butanol layers was observed to become less distinct. The delipidated protein solution was concentrated using an Amicon® Ultra 10 kDa regenerated cellulose (RC) spin concentrator (Millipore). The sample was concentrated by 50% increments, with an equal volume of sample buffer (20 mM Tris-Cl, pH 7.4, 100 mM NaCl, 2 mM DTT) added after each concentration step. The protein solution was thoroughly mixed after each

concentration step to prevent protein precipitation at the membrane. The protein was washed 6–8 times by diluting with sample buffer to 50% of the current volume and reconcentrating. The concentrated protein solution was dialyzed against 1-2 L of sample buffer overnight at 4°C or 22°C, as no significant differences in sample quality were observed between the two temperatures. Following dialysis, the protein was concentrated to 1mg/mL, aliquoted, and stored at -80°C. Protein quality and purity were assessed using SDS-PAGE with Coomassie staining and delipidation was confirmed using protein NMR and X-ray crystallography (Figure S1). ^{1}H-^{15}N HSQC spectra were collected to confirm delipidation[3], using a Bruker AVANCE III 600 MHz instrument equipped with a room temperature QXI-probe. NMR data was processed with NMRPipe and analyzed with the NMRFAM-Sparky distribution.[4,5]

ANS Competition Assay

The binding affinity of 8-Anilinonaphthalene-1-sulfonic acid (ANS) (Cayman Chemical) with FABP4 was determined by mixing 0.5 μM ANS with increasing concentrations of FABP4 (0.01 μM-15 μM) in 300 μL Assay Buffer (50 mM Tris-Cl pH 7.4, 100 mM NaCl, and 2 mM DTT). Samples were incubated for 2 minutes in the dark, and 250 μL of each sample was transferred to a Greiner UV-STAR® 96-well black microplate (Item No.: 655809). Fluorescence measurements were taken in triplicate using a SpectraMax ID5 microplate reader with an endpoint scan at an excitation wavelength of 370 nm and an emission wavelength of 480 nm. All steps were carried out at room temperature. The data were analyzed in GraphPad Prism using nonlinear regression with a quadratic ligand depletion binding model (Figure S2), as described by the equation:

$$Y = D_{max} * \frac{(K_d + x + p) - \sqrt{(K_d + x + p)^2 - 4px}}{2p} + BL$$

Where Y represents the observed fluorescence intensity, and x corresponds to the ligand concentration, in this case, the FABP4 protein concentration (μM). The parameter p is the fixed concentration of ANS held constant at 0.5 μM. D_{max} represents the maximum fluorescence intensity observed when all binding sites are occupied, K_d is the apparent dissociation constant (μM), and BL is the baseline fluorescence in the absence of ligand.

For the binding inhibition assay, a total of 275 μL of Assay Buffer containing 0.5 μM FABP4 and 0.5 μM ANS was mixed with 15 μL of a concentrated per- and polyfluoroalkyl substance (PFAS) or alkanoic acid stock solution. Each PFAS stock was prepared in ethanol or methanol, depending on the compound's solubility. Adding 15 μL of the stock to the assay tube resulted in the desired final ligand concentrations while maintaining a consistent final solvent concentration of 5%. For the 0 μM ligand control, 15 μL of the solvent (without ligand) was added to account for any minor reductions in fluorescence caused by the solvent itself. For the compound with the lowest reported solubility (PFHxDA), the highest

concentration tested was verified by dynamic light scattering (DLS) to confirm the sample was monodisperse and did not contain aggregates.[6] Samples were incubated for 2 minutes in the dark, and 250 µL of each sample was transferred to a Greiner UV-STAR® 96-well black microplate. Fluorescence measurements were taken in triplicate using a SpectraMax ID5 microplate reader with an endpoint scan at an excitation wavelength of 370 nm and an emission wavelength of 480 nm. All steps were carried out at room temperature. The data were analyzed in GraphPad Prism using a sigmoidal four-parameter logistic (4PL) regression model, where x represents the logarithm of the ligand concentration. The model was used to fit the experimental data and determine the IC_{50} values for each tested compound, representing the concentration required to displace 50% of ANS bound to FABP4 (Figure S3-S5).

The IC_{50} values obtained from the binding inhibition assay were converted to K_i values using the IC_{50}-to-K_i converter developed by Cer et al.:[7]

$$K_i = \frac{I_{50}}{\frac{L_{50}}{K_d} + \frac{P_0}{K_d} + 1}$$

where P_0 and L_{50} are free protein and ligand concentrations, respectively. The following parameters were entered into the converter: protein concentration of 0.5 µM, ligand (ANS) concentration of 0.5 µM, and the dissociation constant (K_d) of ANS for FABP4 as 0.508 µM, determined from the earlier assay (Figure S2). The IC_{50} values for each inhibitor were used as the input parameter for inhibition. The calculations were performed using the 'free concentrations' option, which accounts for the unbound fractions of the protein and ligand in the system, and the K_i values were taken from the output under the 'competitive' model. The resulting K_i values represent the binding affinities of the tested PFAS compounds, accounting for the effects of protein and ligand concentrations on the measured IC_{50} values. As the fluorescent probe ANS is well-characterized and binds in a 1:1 stoichiometry with FABP4 (Fig. S2),[8] the calculated K_i values are reported as K_d values to reflect the equilibrium dissociation constants for PFAS binding.

Protein Crystallization

Complexes of FABP4 with PFOA, PFDA, ANS and PFHxDA were prepared by addition of a 10-fold molar excess of the compound to a 1 mg/ml protein solution in 20 mM Tris-HCl, pH 7.4, 100 mM NaCl, 2 mM DTT, and 0.01% Triton-100. Samples were incubated overnight at approximately 20°C with mild shaking. Samples were then concentrated to a final protein concentration of 8-10 mg/mL. Initial crystallization screenings were carried out with a Crystal Gryphon robot (Art Robbins Instruments). The following commercially available crystallization kits were used: SaltRx HT (Hampton Research), Wizard Classic 1 and 2, and Wizard Classic 3 and 4 blocks (Rigaku). Droplets were set up by mixing the protein-ligand complex sample (0.3 µl) with the crystallization reagent (0.3 µl) in a 1:1 ratio. They were then equilibrated against a 58 µl reservoir solution in 96-well sitting-drop INTELLI-PLATES. All plates were

placed in a Gallery Plate Hotel at a constant temperature of 293 K. Crystals appeared in one week under several conditions (K/Na phosphate, Na citrate tribasic, NH4 citrate and PEG-4000) at pH range 6.9-8.5. Attempts to improve the quality and the size of the crystals using the hanging-drop vapor-diffusion method were performed up to the microliter range in 24-well VDX crystallization plates (Hampton Research). High-quality diffraction-grade single crystals of the FABP4-ligand complex were obtained at pH 6.5 using 1.55-1.6 M sodium citrate as the precipitant. FABP4-ANS complex crystallized in either 1.6 M sodium citrate or 30% PEG-4000, 0.2 M Li_2SO_4, 0.1 M Tris-HCl solution at pH 8.5. Apo-FABP4 crystals were grown in 2.5 M ammonium sulfate and 0.1 M Bis-Tris, pH 7.0.

X-ray Data Collection, Phasing, and Model Refinement

For x-ray data collection, crystals were flash-frozen in a liquid nitrogen stream mounted by nylon loops after briefly soaking in a cryoprotectant solution consisting of the mother liquor and 5% glycerol. Diffraction data was collected at 100 K up to 1.24 Å (FABP4-PFOA), 1.4 Å (FABP4-PFDA), 1.35 Å (FABP4-PFHxDA), 1.4 Å (FABP4-ANS) and 1.30 Å (FABP4_Apo) resolutions on in-house copper rotating-anode generator (Rigaku MicroMax-007 HF) equipped with an EIGER R 4M detector. The X-ray data set was processed and scaled with *CrysAlis^{Pro}* 41.64.122a (Rigaku) and the CCP4 suite[9] of programs. Diffraction data for FABP4-PFOA and FABP4-PFDA complex crystals were indexed in space group C2 with one molecule in the asymmetric unit. FABP4-PFHxDA, FABP4-ANS, and FABP4_Apo crystals were indexed in space group P212121 with one molecule in the asymmetric unit. The unit cell dimensions are reported in Table S3. The structure was determined by molecular replacement using Phaser[10] from the PHENIX suite.[11] The starting model was the structure of the human adipocyte fatty acid-binding protein (PDB entry 7WC3). Auto model building resulted in an initial model of 129 amino-acid residues built with R_{work} 18.89% and R_{free} 20.54% (FABP4-PFOA complex), R_{work} 23.54% and R_{free} 29.23% (FABP4-PFDA complex), R_{work} 24.36% and R_{free} 26.68% (FABP4-PFHxDA complex), R_{work} 27.97% and R_{free} 30.80% (FABP4-ANS complex) and R_{work} 27.13% and R_{free} 29.87% (FABP4_Apo). The structures were refined with Phenix along with manual model building using Coot. The difference Fourier map revealed the presence of bound ligands in the active site of the FABP4. Ligand bound at the active site was clearly defined in the electron density map. Ligand was autofit into the electron density using the LigandFit procedure within Phenix, followed by manual rotation of CF2 groups using chi angles rotation mode in Coot.[12] The structures were refined to 1.24 Å resolutions with a final R_{work} of 12.44% and R_{free} of 15.70% (FABP4-PFOA complex), 1.4 Å resolution with a final R_{work} of 13.96% and R_{free} of 19.74% (FABP4-PFDA complex), 1.35 Å resolution with a final R_{work} of 17.81% and R_{free} of 21.10% (FABP4-PFHxDa complex), 1.40 Å resolution with a final R_{work} 18.58% and R_{free} 20.50% (FABP4-ANS complex) and 1.30 Å resolution with a final R_{work} 18.05% and R_{free} 19.29% (FABP4_Apo). Data collection and refinement statistics are

summarized in Table S3. The atomic coordinates and structure factors for FABP4_Apo and FABP4 bound to PFOA, PFDA, PFHxDA, and ANS have been deposited in the Protein Data Bank (PDB IDs: 9OB7, 9MIW, 9MIZ, 9MP2, and 9OB8 respectively).

Table S1: Experimentally Determined PFAS-Bound Protein Structures Available on the Protein Data Bank (PDB)

**Protein Name**	**PFAS Molecule**	**PDB Entry ID**	**Reference**
Human Serum Albumin	Perfluorooctanesulfonic Acid (PFOS)	4E99	[13]
Human Serum Albumin	Perfluorooctanoic Acid (PFOA)	7AAI	[14]
Human Serum Albumin	GenX	7Z57	[15]
Human Transthyretin	Perfluorooctanoic Acid (PFOA)	5JID	[16]
Human Transthyretin	Perfluorooctanesulfonic Acid (PFOS)	5JIM	[16]
Sea Bream Transthyretin	Perfluorooctanoic Acid (PFOA)	6GON	[17]
Sea Bream Transthyretin	Perfluorooctanoic Acid (PFOA)	6GOO	[17]
Human Heart Fatty Acid-Binding Protein	Perfluoroheptanoic Acid (PFHpA)	7FD7	(To be published)
Human Heart Fatty Acid-Binding Protein	Perfluorooctanoic Acid (PFOA)	7FEK	(To be published)
Human Heart Fatty Acid-Binding Protein	Perfluorononanoic Acid (PFNA)	7FEU	(To be published)
Human Peroxisome Proliferator-Activated Receptor Gamma (PPARγ) Ligand Binding Domain (LBD)	Perfluorooctanoic Acid (PFOA)	8U57	[18]

Table S2: FABP4 binding affinities of PFCAs, alkanoic acids, and PFAS with sulfonic acid, sulfonamide, ether, and alcohol headgroups. IC_{50} values represent the average from triplicate measurements with standard errors reported. The K_i values were calculated as described in Cer et al.,[7] reported with their associated standard errors. The abbreviation "ND" indicates that no binding was detected.

C-chain	Perfluorocarboxylic Acids (PFCA)	IC_{50} (μM) ± SD	r^2	K_i (μM) ± error
4	Perfluorobutanoic Acid (PFBA)	2843 ± 140	0.9981	1181 ± 58
5	Perfluoropentanoic Acid (PFPeA)	792.8 ± 35.6	0.9972	329.2 ± 14.8
6	Perfluorohexanoic Acid (PFHxA)	208.9 ± 10.4	0.9996	86.65 ± 4.29
7	Perfluoroheptanoic Acid (PFHpA)	11.02 ± 0.44	0.9987	4.458 ± 0.178
8	Perfluorooctanoic Acid (PFOA)	4.669 ± 0.13	0.9994	1.82 ± 0.051
9	Perfluorononanoic Acid (PFNA)	5.944 ± 0.33	0.9980	2.35 ± 0.13
10	Perfluorodecanoic Acid (PFDA)	3.537 ± 0.09	0.9984	1.35 ± 0.034
11	Perfluoroundecanoic Acid (PFUnDA)	2.92 ± 0.12	0.9988	1.094 ± 0.045
12	Perfluorododecanoic Acid (PFDoA)	1.613 ± 0.057	0.9976	0.551 ± 0.020
13	Perfluorotridecanoic Acid (PFTrDA)	2.12 ± 0.072	0.9973	0.757 ± 0.026
14	Perfluorotetradecanoic Acid (PFTeDA)	2.002 ± 0.055	0.9981	0.7126 ± 0.020
16	Perfluorohexadecanoic Acid (PFHxDA)	3.446 ± 0.050	0.9987	1.312 ± 0.019
**C-chain**	**Alkanoic Acids**	**IC_{50} (μM) ± SD**	**r^2**	**K_i (μM) ± error**
4	Butanoic Acid	ND	–	ND
5	Pentanoic Acid	ND	–	ND
6	Hexanoic Acid	ND	–	ND
7	Heptanoic Acid	348.1 ± 7.47	0.9983	144.5 ± 3.10
8	Octanoic Acid	32.73 ± 1.72	0.9979	13.48 ± 0.71
9	Nonanoic Acid	29.95 ± 0.61	0.9989	12.32 ± 0.25
10	Decanoic Acid	3.386 ± 0.136	0.9981	1.287 ± 0.052
11	Undecanoic Acid	2.249 ± 0.078	0.9978	0.8152 ± 0.028
12	Dodecanoic Acid	2.277 ± 0.072	0.9979	0.8268 ± 0.026
13	Tridecanoic Acid	3.459 ± 0.137	0.9985	1.318 ± 0.052
14	Tetradecanoic Acid	1.341 ± 0.055	0.9971	0.4381 ± 0.018
16	Hexadecanoic Acid	1.905 ± 0.082	0.9981	0.6723 ± 0.029
**C-chain**	**PFAS with Alternate Headgroups**	**IC_{50} (μM) ± SD**	**r^2**	**K_i (μM) ± error**
8	Perfluorooctanesulfonic Acid (PFOS)	11.13 ± 0.53	0.999	4.504 ± 0.214
8	Perfluorooctanesulfonamide (PFOSA)	17.02 ± 0.93	0.998	6.95 ± 0.38
6	Hexafluoropropylene Oxide Dimer Acid (HFPO-DA)	47.32 ± 2.65	0.998	19.53 ± 1.09
10	8:2 Fluorotelomer Sulfonic Acid (8:2 FtS)	38.35 ± 2.04	0.999	15.81 ± 0.84
10	1H,1H,2H,2H-Perfluoro-1-decanol (8:2 FTOH)	ND	–	ND

Table S3: X-ray crystal data collection and refinement statistics.

	**FABP4-PFOA**	**FABP4-PFDA**	**FABP4-PFHxDA**	**FABP4-ANS**	**FABP4-Apo**
Space group	C2	C2	P 212121	P212121	P212121
Unit-cell *a, b, c* (Å)	118.752, 37.716, 28.546, β = 93.02(°)	118.746, 37.709, 28.490 β = 92.50(°)	32.217, 53.564, 75.216	32.837, 53.744, 74.844	32.319, 53.617, 74.684
Resolution (Å)<br><br>Total reflections	22.51–1.24 (1.26 –1.24)<br>505153 (2913)	27.29–1.40 (1.42–1.40)<br>222985 (6098)	27.61-1.35 (1.37-1.35)<br>250919 (7717)	26.24-1.40 1.42-1.40<br>257022 (6260)	25.95-1.30 (1.32-1.30)<br>337962 (4177)
Unique reflections	35429 (1325)	24826 (1205)	29408 (1430)	26876 (1314)	32446 (1367)
Redundancy	14.3 (2.2)	9.0 (5.1)	8.5 (5.4)	9.6 (4.8)	10.4 (3.1)
Completeness (%)	98.7 (77.3)	99.4 (95.9)	100.0 (100.0)	1000 (99.9)	99.3 (87.8)
Average I/σ(I)	52.8 (6.9)	13.4 (1.9)	25.5 (2.2)	22.8 (1.8)	24.8 (2.0)
R_{merge} (%)[a]	2.7 (9.0)	8.5 (83.6)	4.6 (79.7)	6.0 (95.2)	5.3 (58.6)
Refinement Statistics					
Resolution (Å)	22.51-1.24 (1.27-1.24)	22.46-1.40 (1.46-1.40)	27.61-1.35 (1.39-1.35)	24.68-140 (1.46-1.40)	25.23-1.30 (1.34-1.30)
No. of reflections	35428 (2266)	24819 (2643)	29350 (2894)	26812 (2912)	32389 (2667)
R_{work} (%)	12.44 (12.44)	13.96 (20.49)	17.81 (20.96)	18.58 (27.37)	18.05 (24.29)
R_{free} (%)[b]	15.70 (16.89)	19.74 (29.22)	21.10 (24.30)	20.50 (27.76)	19.29 (26.46)
R.m.s.d. bonds (Å)	0.006	0.006	0.005	0.006	0.005
R.m.s.d. angles (°)	1.114	0.950	0.880	0.794	0.839
Dihedral angles					
Most favored (%)	98.5	99.2	96.9	97.0	96.9
Allowed (%)	1.5	0.8	3.1	3.0	3.1
Average B (Å^2) / atoms					
All atoms	13.62	18.70	21.57	17.35	13.92
Protein<br>Ligand<br>Solvent<br>Number of non-hydrogen atoms	10.12<br>34.13<br>25.49<br>1399	15.41<br>67.64<br>28.36<br>1334	13.70<br>157.24<br>28.51<br>1289	14.58<br>23.08<br>28.05<br>1337	11.26<br>14.05<br>25.95<br>1266
Macromolecules<br>Ligands<br>Water	1108<br>50<br>241	1089<br>31<br>214	1030<br>49<br>210	1051<br>31<br>255	1029<br>10<br>227
Protein residues	134	134	131	134	131
PDB code	9MIW	9MIZ	9MP2	9OB8	9OB7

[a]$R_{merge} = \Sigma_{hkl}\Sigma_i|I_i(hkl) - \langle I(hkl)\rangle|/\Sigma_{hkl}\Sigma_i I_i(hkl)$. [b]$R_{free}$ was calculated from 5% randomly selected reflection for cross-validation. All other measured reflections were used during refinement.

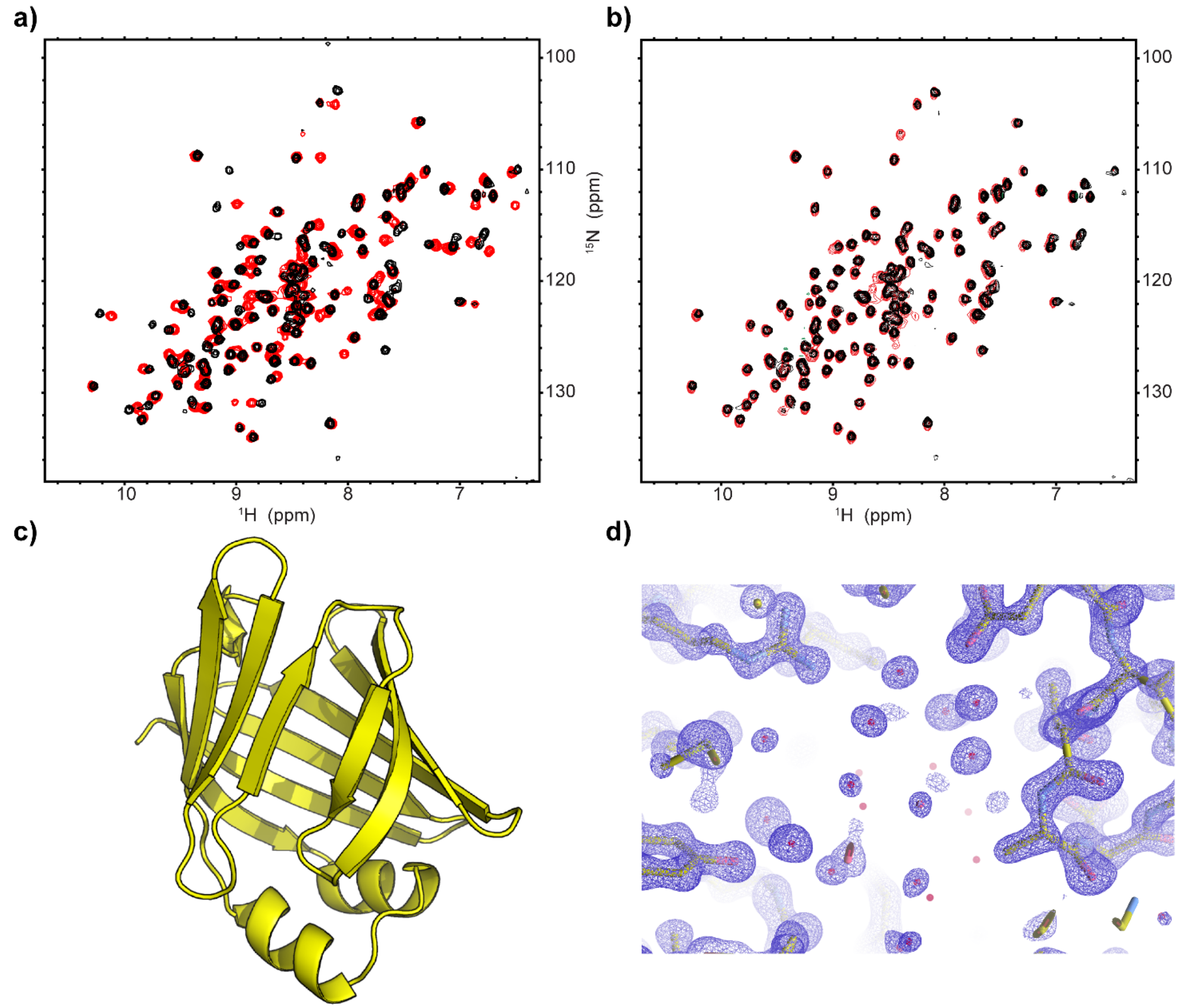

Figure S1. ^{1}H-^{15}N HSCQ spectra of human FABP4 before (black) and after (red) delipidation by butanol extraction. Spectra were collected at 25°C with a 600 MHz instrument. b) ^{1}H-^{15}N HSCQ spectra of FABP4 pre-delipidation (black) and delipidated FABP4 reconstituted with palmitate (red), demonstrating restoration of spectral features consistent with a lipid-bound state. c) Ribbon diagram of Apo-FABP4 (PDB ID: 9OB7), showing the canonical β-barrel structure and portal helix region. d) 2Fo–Fc electron density map (σ = 2.0) of the Apo-FABP4 binding cavity highlighting a lack of density apart from ordered solvent molecules. Several water molecules are resolved within the cavity and occupy positions previously shown to be conserved across the fatty acid binding protein (FABP) family.[19,20]

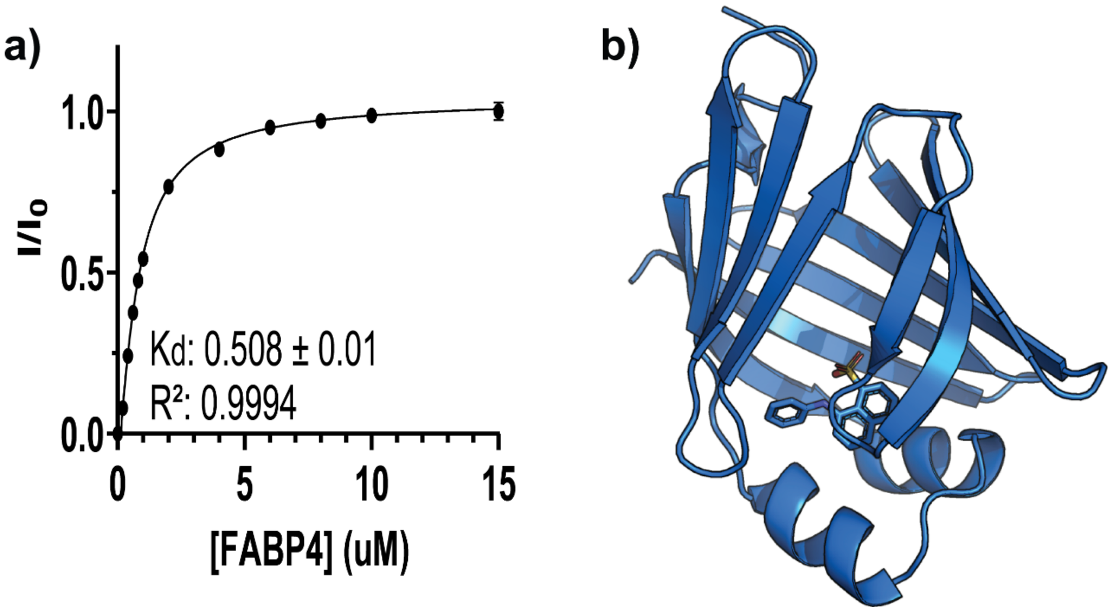

Figure S2. Binding affinity of ANS for FABP4 and structure of the FABP4-ANS complex. **a)** Graph showing the normalized fluorescence intensity of FABP4 (0.01 µM to 15 µM). Data points represent triplicate measurements, with error bars indicating the standard deviation. Nonlinear regression was performed using a Quadratic Ligand Depletion Binding Model, yielding a dissociation constant (K_d) of 0.508 µM for the FABP4-ANS interaction. b) Crystal structure of FABP4 bound to ANS (PDB: 9OB8), indicating binding to the canonical, lipid binding pocket. ANS is depicted in sticks.

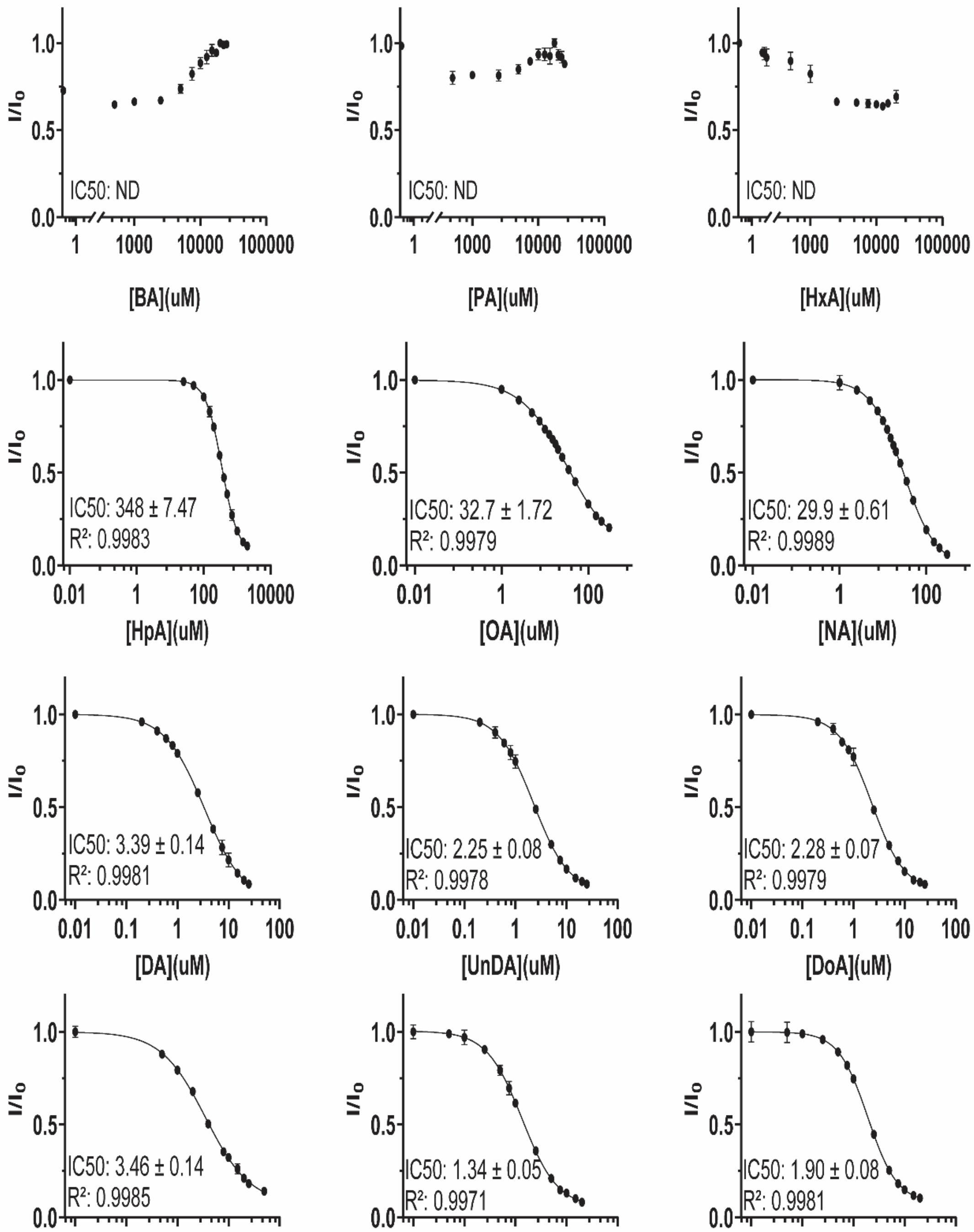

Figure S3. IC_{50} curves of alkanoic acids binding to FABP4 measured by displacement of ANS from FABP4. Each curve represents the normalized fluorescence intensity relative to the control (0 µM ligand), plotted against the log concentration of the ligand. IC_{50} values were determined using a four-parameter logistic (4PL) regression model in GraphPad Prism. Data points represent the mean of triplicate measurements, and error bars indicate the standard deviation.

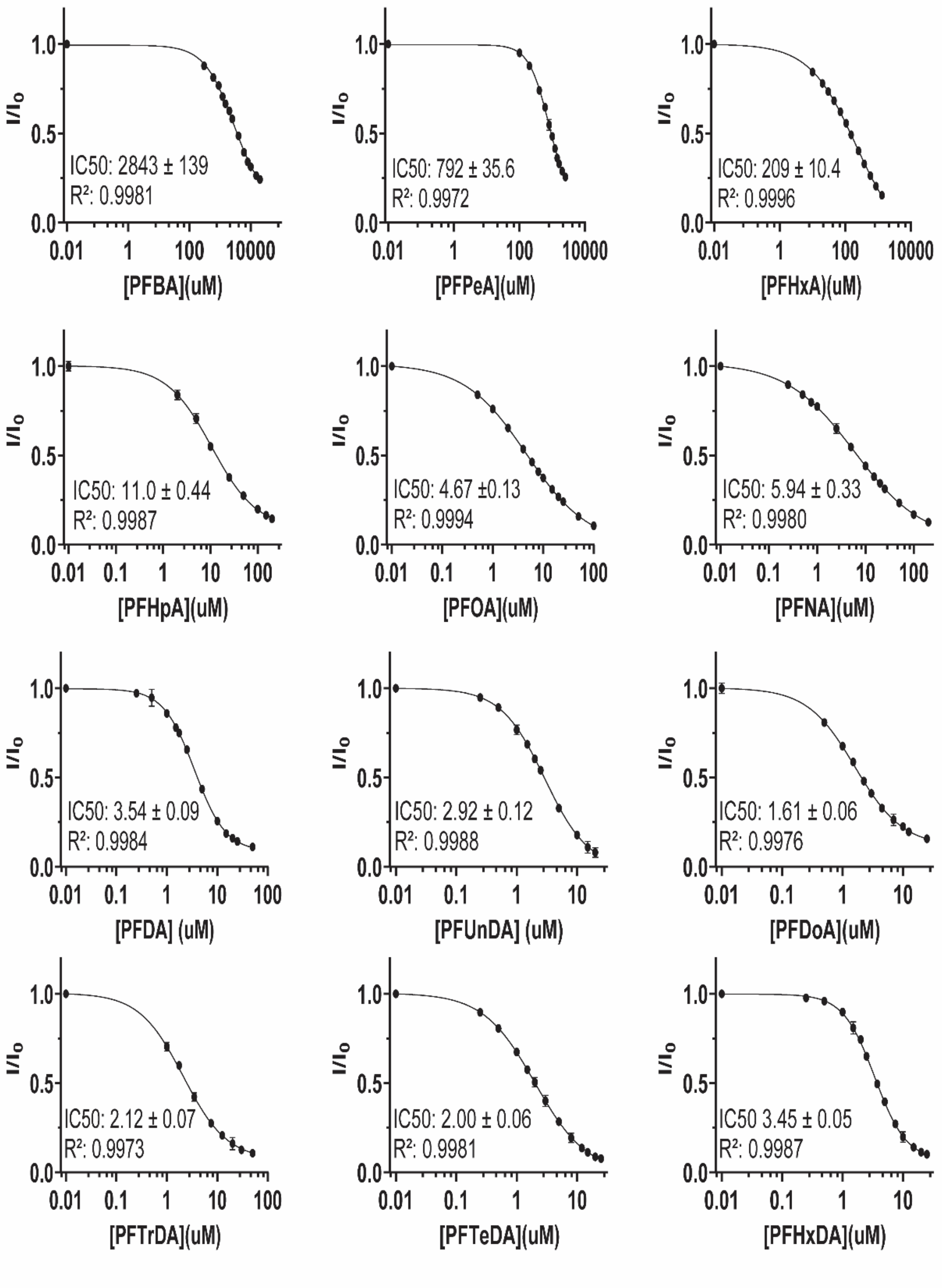

Figure S4. IC_{50} curves of perfluorinated carboxylic acids (PFCAs) binding to FABP4, measured by displacement of ANS from FABP4. The curves display the normalized fluorescence intensity relative to the control (0 μM ligand) plotted against the log concentration of the ligand. IC_{50} values were calculated using a 4PL regression model. Data represent triplicate measurements, with error bars showing the standard deviation.

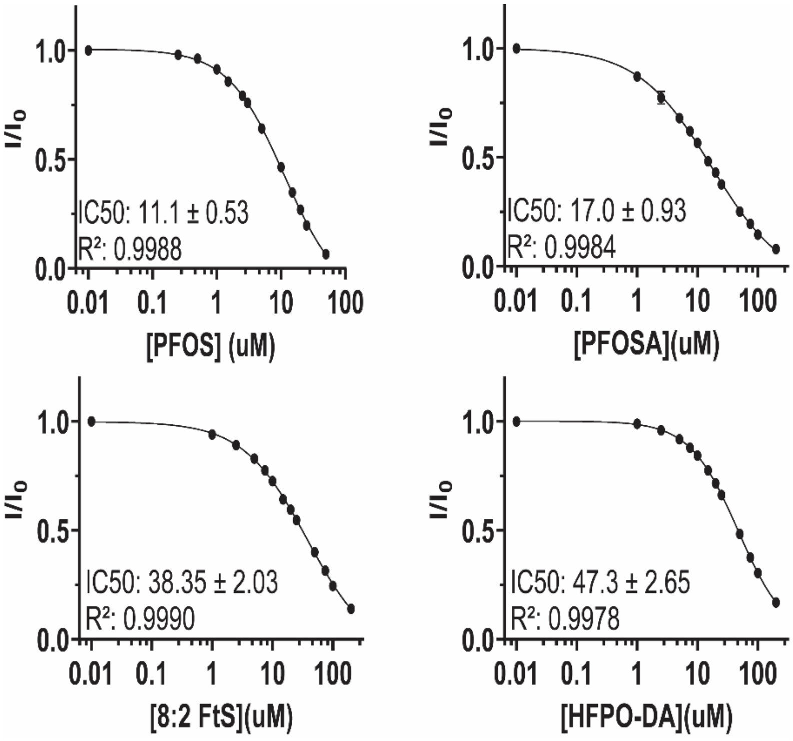

Figure S5. IC_{50} curves of other PFAS compounds binding to FABP4. IC_{50} curves were measured by displacement of ANS from FABP4 by additional PFAS compounds, including perfluorooctanesulfonic acid (PFOS), perfluorooctanesulfonamide (PFOSA), hexafluoropropylene oxide dimer acid (HFPO-DA), and 8:2 fluorotelomer sulfonic acid (8:2 FtS). Normalized fluorescence intensity relative to the control (0 µM ligand) is plotted against the log concentration of each compound. IC_{50} values were determined using a 4PL regression model, with data shown as the mean of triplicate measurements and error bars indicating standard deviation.

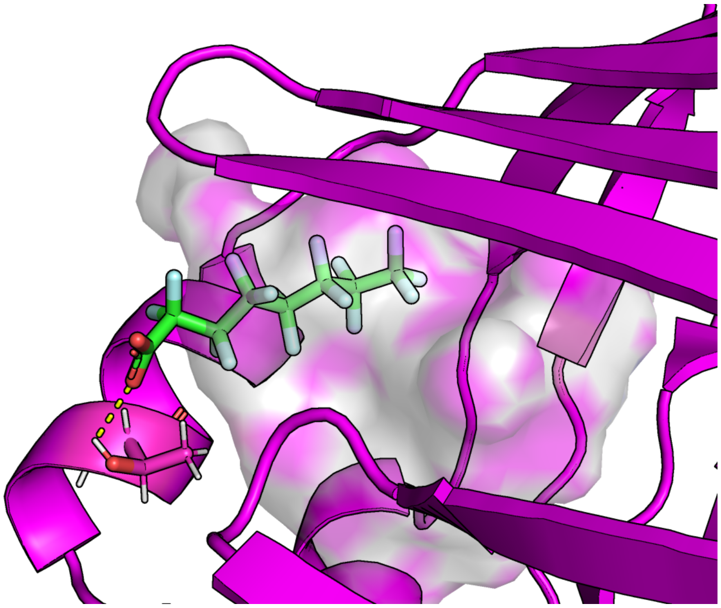

Figure S6. Zoom of the crystal structure of PFOA bound to FAPB4 in the secondary binding site (PDB: 9MIW). The protein cavity is depicted as a semi-transparent surface, colored according to atomic identity: gray for hydrogens, blue for nitrogens, and red for oxygens, with carbons magenta. PFOA and Thr29 are depicted as sticks, with their hydrogen-bond highlighted with a dashed yellow line. For clarity, PFOA bound in the primary site is omitted.

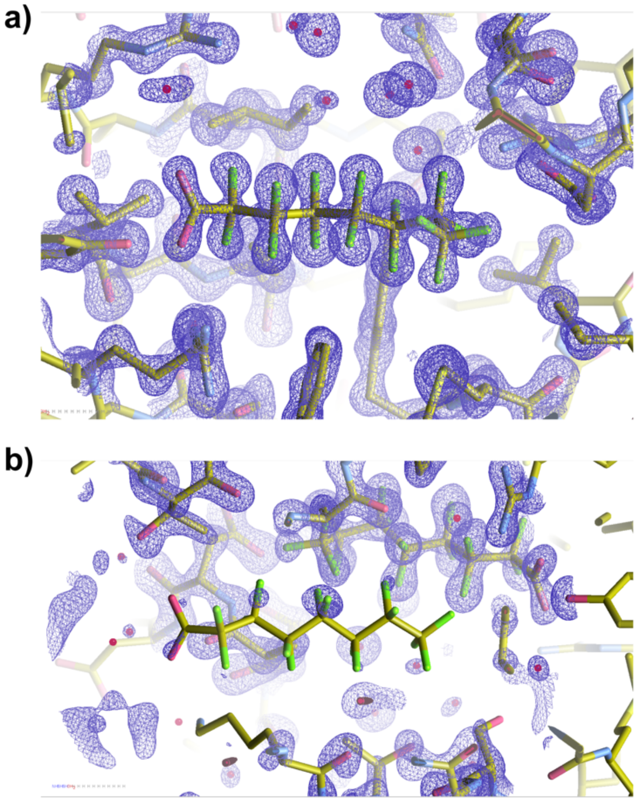

Figure S7. Electron density 2Fo − Fc maps with contour level σ = 2.0 of PFOA in the (a) primary and (b) secondary binding sites.

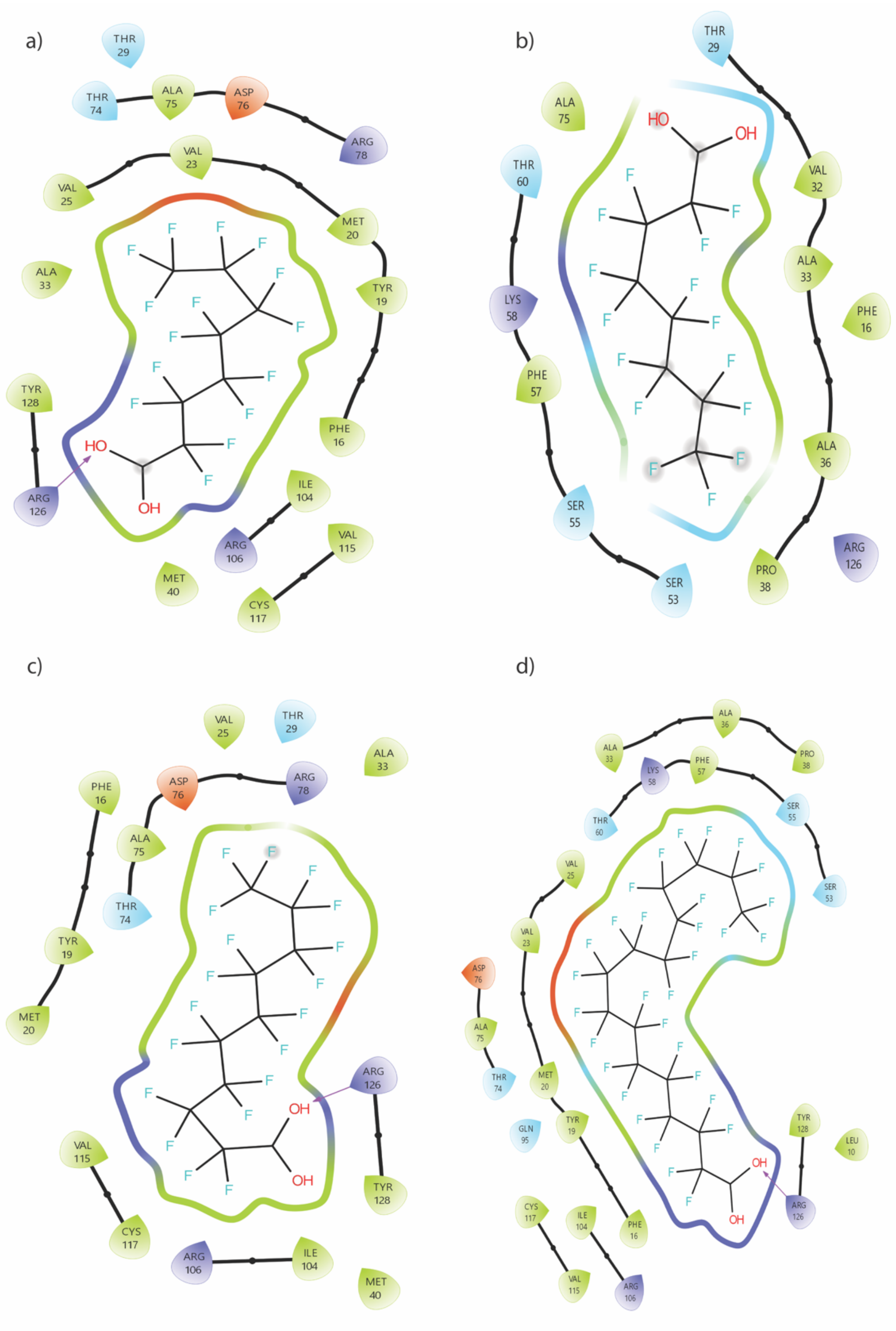

Figure S8: Ligand interaction diagrams of FABP4 with PFOA (primary binding site) (a), PFOA (secondary binding site) (b), PFDA (c), and PFHxDA (d). Residues within 4 Å of the ligand include hydrophobic residues (green), positively charged residues (purple), negatively charged residues (red), and polar residues (light blue). Hydrogen bonds are indicated by purple arrows, and black lines denote residues on the same structural element (loop, β-sheet, or α-helix). Images were generated using Maestro Viewer.[21]

Supplemental References

(1) Azatian, S. B.; Kaur, N.; Latham, M. P. Increasing the Buffering Capacity of Minimal Media Leads to Higher Protein Yield. *J Biomol NMR* 2019, *73* (1–2), 11–17. https://doi.org/10.1007/s10858-018-00222-4.

(2) Marr, E.; Tardie, M.; Carty, M.; Brown Phillips, T.; Wang, I.-K.; Soeller, W.; Qiu, X.; Karam, G. Expression, Purification, Crystallization and Structure of Human Adipocyte Lipid-Binding Protein (AP2). *Acta Crystallogr Sect F Struct Biol Cryst Commun* 2006, *62* (11), 1058–1060. https://doi.org/10.1107/S1744309106038656.

(3) Wang, Q.; Rizk, S.; Bernard, C.; Lai, M. P.; Kam, D.; Storch, J.; Stark, R. E. Protocols and Pitfalls in Obtaining Fatty Acid-Binding Proteins for Biophysical Studies of Ligand-Protein and Protein-Protein Interactions. *Biochem Biophys Rep* 2017, *10*, 318–324. https://doi.org/10.1016/j.bbrep.2017.05.001.

(4) Lee, W.; Tonelli, M.; Markley, J. L. NMRFAM-SPARKY: Enhanced Software for Biomolecular NMR Spectroscopy. *Bioinformatics* 2015, *31* (8), 1325–1327. https://doi.org/10.1093/bioinformatics/btu830.

(5) Delaglio, F.; Grzesiek, S.; Vuister, GeertenW.; Zhu, G.; Pfeifer, J.; Bax, A. NMRPipe: A Multidimensional Spectral Processing System Based on UNIX Pipes. *J Biomol NMR* 1995, *6* (3). https://doi.org/10.1007/BF00197809.

(6) Allen, S. J.; Dower, C. M.; Liu, A. X.; Lumb, K. J. Detection of Small-Molecule Aggregation with High-Throughput Microplate Biophysical Methods. *Curr Protoc Chem Biol* 2020, *12* (1). https://doi.org/10.1002/cpch.78

(7) Cer, R. Z.; Mudunuri, U.; Stephens, R.; Lebeda, F. J. IC50-to-Ki: A Web-Based Tool for Converting IC50 to Ki Values for Inhibitors of Enzyme Activity and Ligand Binding. *Nucleic Acids Res* 2009, *37* (Web Server issue), W441-5. https://doi.org/10.1093/nar/gkp253.

(8) Ory, J. J.; Banaszak, L. J. Studies of the Ligand Binding Reaction of Adipocyte Lipid Binding Protein Using the Fluorescent Probe 1, 8-Anilinonaphthalene-8-Sulfonate. *Biophys J* 1999, *77* (2), 1107–1116. https://doi.org/10.1016/S0006-3495(99)76961-4.

(9) Winn, M. D.; Ballard, C. C.; Cowtan, K. D.; Dodson, E. J.; Emsley, P.; Evans, P. R.; Keegan, R. M.; Krissinel, E. B.; Leslie, A. G. W.; McCoy, A.; McNicholas, S. J.; Murshudov, G. N.; Pannu, N. S.; Potterton, E. A.; Powell, H. R.; Read, R. J.; Vagin, A.; Wilson, K. S. Overview of the *CCP* 4 Suite and Current Developments. *Acta Crystallogr D Biol Crystallogr* 2011, *67* (4), 235–242. https://doi.org/10.1107/S0907444910045749.

(10) McCoy, A. J.; Grosse-Kunstleve, R. W.; Adams, P. D.; Winn, M. D.; Storoni, L. C.; Read, R. J. *Phaser* Crystallographic Software. *J Appl Crystallogr* 2007, *40* (4), 658–674. https://doi.org/10.1107/S0021889807021206.

(11) Adams, P. D.; Afonine, P. V.; Bunkóczi, G.; Chen, V. B.; Echols, N.; Headd, J. J.; Hung, L.-W.; Jain, S.; Kapral, G. J.; Grosse Kunstleve, R. W.; McCoy, A. J.; Moriarty, N. W.; Oeffner, R. D.; Read, R. J.; Richardson, D. C.; Richardson, J. S.; Terwilliger, T. C.; Zwart, P. H. The Phenix Software for Automated Determination of Macromolecular Structures. *Methods* 2011, *55* (1), 94–106. https://doi.org/10.1016/j.ymeth.2011.07.005.

(12) Emsley, P.; Lohkamp, B.; Scott, W. G.; Cowtan, K. Features and Development of *Coot*. *Acta Crystallogr D Biol Crystallogr* 2010, *66* (4), 486–501. https://doi.org/10.1107/S0907444910007493.

(13) Luo, Z.; Shi, X.; Hu, Q.; Zhao, B.; Huang, M. Structural Evidence of Perfluorooctane Sulfonate Transport by Human Serum Albumin. *Chem Res Toxicol* 2012, *25* (5), 990–992. https://doi.org/10.1021/tx300112p.

(14) Maso, L.; Trande, M.; Liberi, S.; Moro, G.; Daems, E.; Linciano, S.; Sobott, F.; Covaceuszach, S.; Cassetta, A.; Fasolato, S.; Moretto, L. M.; De Wael, K.; Cendron, L.; Angelini, A. Unveiling the Binding Mode of Perfluorooctanoic Acid to Human Serum Albumin. *Protein Science* 2021, *30* (4), 830–841. https://doi.org/10.1002/pro.4036.

(15) Moro, G.; Liberi, S.; Vascon, F.; Linciano, S.; De Felice, S.; Fasolato, S.; Foresta, C.; De Toni, L.; Di Nisio, A.; Cendron, L.; Angelini, A. Investigation of the Interaction between Human Serum Albumin and Branched Short-Chain Perfluoroalkyl Compounds. *Chem Res Toxicol* 2022, *35* (11), 2049–2058. https://doi.org/10.1021/acs.chemrestox.2c00211.

(16) Zhang, J.; Begum, A.; Brännström, K.; Grundström, C.; Iakovleva, I.; Olofsson, A.; Sauer-Eriksson, A. E.; Andersson, P. L. Structure-Based Virtual Screening Protocol for *in Silico* Identification of Potential Thyroid Disrupting Chemicals Targeting Transthyretin. *Environ Sci Technol* 2016, *50* (21), 11984–11993. https://doi.org/10.1021/acs.est.6b02771.

(17) Zhang, J.; Grundström, C.; Brännström, K.; Iakovleva, I.; Lindberg, M.; Olofsson, A.; Andersson, P. L.; Sauer-Eriksson, A. E. Interspecies Variation between Fish and Human Transthyretins in Their Binding of Thyroid-Disrupting Chemicals. *Environ Sci Technol* 2018, *52* (20), 11865-11874. https://doi.org/10.1021/acs.est.8b03581

(18) Pederick, J. L.; Frkic, R. L.; McDougal, D. P.; Bruning, J. B. A Structural Basis for the Activation of Peroxisome Proliferator-Activated Receptor Gamma (PPARγ) by Perfluorooctanoic Acid (PFOA). *Chemosphere* 2024, *354*, 141723. https://doi.org/10.1016/j.chemosphere.2024.141723.

(19) Likić, V. A.; Juranić, N.; Macura, S.; Prendergast, F. G. A "Structural" Water Molecule in the Family of Fatty Acid Binding Proteins. *Protein Sci* 2000, *9* (3), 497–504. https://doi.org/10.1110/ps.9.3.497.

(20) Wiesner, S.; Kurian, E.; Prendergast, F. G.; Halle, B. Water Molecules in the Binding Cavity of Intestinal Fatty Acid Binding Protein: Dynamic Characterization by Water17O and 2H Magnetic Relaxation Dispersion. *J Mol Biol* 1999, *286* (1), 233–246. https://doi.org/10.1006/jmbi.1998.2490.

(21) Schrödinger, L. Maestro, Schrödinger Release 2024-4. Schrödinger, LLC: New York, NY 2024.
